# Applicability of the theory of planned behavior in explaining the general practitioners eLearning use in continuing medical education

**DOI:** 10.1186/s12909-016-0738-6

**Published:** 2016-08-22

**Authors:** Arash Hadadgar, Tahereh Changiz, Italo Masiello, Zahra Dehghani, Nahidossadat Mirshahzadeh, Nabil Zary

**Affiliations:** 1 Department of Learning, Informatics, Management and Ethics, Karolinska Institutet, 17177 Stockholm, Sweden; 2 Medical education research center, Isfahan University of medical sciences, Hezar Jerib Av, Isfahan, Iran; 3 Medical education department, Isfahan University of medical sciences, Hezar Jerib Av, Isfahan, Iran; 4 Department of Clinical Science and Education, Karolinska Institutet, Södersjukhuset, 11883 Stockholm, Sweden; 5 Continuing medical education office, Isfahan University of medical sciences, Isfahan, Iran

**Keywords:** Continuing medical education, e-learning, Theory of planned behavior, General practitioner

## Abstract

**Background:**

General practitioners (GP) update their knowledge and skills by participating in continuing medical education (CME) programs either in a traditional or an e-Learning format. GPs’ beliefs about electronic format of CME have been studied but without an explicit theoretical framework which makes the findings difficult to interpret. In other health disciplines, researchers used theory of planned behavior (TPB) to predict user’s behavior.

**Methods:**

In this study, an instrument was developed to investigate GPs’ intention to use e-Learning in CME based on TPB. The goodness of fit of TPB was measured using confirmatory factor analysis and the relationship between latent variables was assessed using structural equation modeling.

**Results:**

A total of 148 GPs participated in the study. Most of the items in the questionnaire related well to the TPB theoretical constructs, and the model had good fitness. The perceived behavioral control and attitudinal constructs were included, and the subjective norms construct was excluded from the structural model. The developed questionnaire could explain 66 % of the GPs’ intention variance.

**Conclusions:**

The TPB could be used as a model to construct instruments that investigate GPs’ intention to participate in e-Learning programs in CME. The findings from the study will encourage CME managers and researchers to explore the developed instrument as a mean to explain and improve the GPs’ intentions to use eLearning in CME.

**Electronic supplementary material:**

The online version of this article (doi:10.1186/s12909-016-0738-6) contains supplementary material, which is available to authorized users.

## Background

Given the fast pace with which knowledge expands and technology develops, updating the knowledge and skills of physicians and other health professionals is necessary in order to ensure that the care they provide is based on the latest medical evidence. Therefore, in an increasing number of countries [1, 2], including middle-income countries such as Iran [3], continuing medical education (CME) is mandatory for all practicing physicians. The use of e-Learning in CME (eCME) could improve accessibility, increase flexibility and provide rapid response to public health needs on a large scale [1, 4]. Additional aspects that contributes to the increased potential of eCME are the digitization of information and emerging new generations of digital native learners [5].

eLearning in CME is rapidly growing [6], but physicians seems to have different beliefs about this form of training. While some are very positive about using e-Learning for CME [7, 8], others still prefer a more traditional training approach [9]. Researchers have investigated the physicians’ concerns in eCME from a barrier perspective [10]. As a scalable and cost-effective way of training, it is therefore worth exploring the physicians’ intention to use eCME and its main related factors.

General practitioners (GP) hold a crucial role in the healthcare as the first formal instance for a medical consultation. For this reason, GPs are the subjects of this study. An increased understanding of their eCME use behaviors would help improve their training and optimize the eCME delivery systems. Icek Ajzen developed the theory of planned behavior (TPB) that can describe and predict behaviors and intentions [11]. This theory has been applied to various fields, such as advertising, public relations and healthcare [11]. It can explain individuals’ behavior of the new technology adoption quite well; not only does it describe the relationship between constructs, but also it helps uncover specific factors that can affect the adoption or use of technology [12]. Other researchers have used this theory for measuring perceived barriers to completing an e-learning program on evidence-based medicine [10], student intention to adopt mobile learning [13], and also acceptance of a software in an undergraduate curriculum [14]. TPB can deliver specific information about users which is needed to conduct improvements [15].

The aim of this study was to explore a theory-driven approach by applying TPB in the construction of a questionnaire and then evaluate its ability to model and explain GPs’ intention to use e-Learning in CME. The rationale of this study is to understand which factors influence GPs’ intention to use eCME in order to provide sustainable eCME for GPs and possibly other populations of users.

To achieve the stated aim, the study addressed the following research questions:

- How do factors extracted by exploratory factor analysis match the theoretical constructs of TPB?
- How does the statistical model of GPs’ intentions fit the data collected from an eCME setting?
- How does the model map GPs’ intention to use eCME?

## Methods

The aim was addressed by first creating a questionnaire designed on TPB principles, testing whether the questionnaire measured its behavioral constructs, and finally modelling GPs intentions.

### Context of study

The CME program at the Isfahan University of Medical Sciences (IUMS) was used as the context of study and representative for the contexts where CME is mandatory for all physicians and with particularities relevant to the middle-income countries in terms of access to technology.

Most GPs participate in face-to-face CME onsite seminars/workshops, or e-CME programs in order to update their knowledge and earn the required credits. GPs can acquire up to 50 % of their CME credits via eCME. The most common type of eCME is based on CD-ROMs, the format of the eCME in this study, which is offline and does not support learner interactions. The GP pays to participate in seminars or eCME. Corporate sponsorship is not allowed in Iran.

### Using the theory of planned behavior to study the eCME users’ intentions

**Fig. 1:**
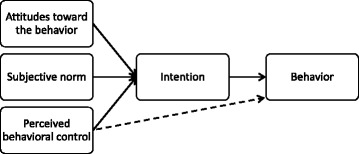
The constructs of the theory of planned behavior

Discovering the human behavior and its dynamics had been one of the concerns of science. In TPB, behavioral intentions are a proxy for real behavior, which are determined by a combination of a person’s attitudes, subjective norms and perceived behavioral control (Fig. 1).

Generally, the more positive the attitude, subjective norms and perceived behavioral control towards a specific behavior are, the stronger the person’s intention to perform it.

#### Elicitation study

The very first step with TPB is to discover participants’ beliefs about the subject matter which is usually called “elicitation study”. In our project, an open-ended questionnaire (about positive and negative dimensions of the TPB constructs) was distributed by two authors (ZD and NM) among physicians in CME seminars. Twenty-three GPs filled the questionnaires. Afterwards, a psychometrist (ZD) did a structures interview with five of these GPs who volunteered and were asked about their intentions to use e-Learning in CME and related factors. Based on the participants’ beliefs, we developed the final questionnaire.

We technically defined the four constructs of the study as follows.

- **Intention (the outcome variable)**: Ajzen offers a generic definition of ‘intention’: “a person’s readiness to perform a given behavior” [16]. If a positive relationship between intention and actual behavior is confirmed, it can be a proxy of actual behavior in the absence of a true measurement [17]. This construct differs from “eLearning readiness” that some researchers focus on. [18]
- **Attitude**: defined as the degree to which an individual favors the examined behavior. We used two groups of factors that influence one’s decision about using technology: perceived usefulness and perceived ease-of-use (the degree to which a person believes that using a particular system would enhance his or her job performance and would be free from effort, respectively). Technologies that are perceived to be less complex to use have a higher possibility of acceptance and use by potential users.
- **Subjective norms**: refer to social pressures that make an individual perform a particular behavior [11]. Different social groups might have different opinions regarding the adoption of a particular technology [12]. For this study, three groups were considered: superiors, CME office staff and peers (physicians). We didn’t include patients and important relatives, because GPs didn’t mention them in the elicitation phase.
- **Perceived behavioral control (PBC)**: accounts for situations where individuals do not have complete control over their behavior, and it has two components [19]. The first is self-efficacy, reflecting personal comfort with using technology, and the other component is controllability, representing the availability of resources needed to use the technology, such as time, money and equipment. The absence of facilitating conditions can negatively impact the intention to usage technology [12].

### Development of the questionnaire

In TPB, the target behavior should be clearly defined. We defined the use of e-learning in a CME context as a formal learning experience, which provides learning material mainly in the form of CD-ROMs. Based on the behavioral components of the TPB, we defined the target behavior under study as ‘Using (ACTION) eLearning (TARGET) for CME (CONTEXT) in the past year (TIME)’. Then the authors followed the recommended standard steps [20] to create the questionnaire.

#### Content and face validity

A standard TPB questionnaire includes items used to measure all of its related constructs. From the elicitation study, we developed the questionnaire and after ensuring that we had at least three items for each construct of the TPB, we then checked the questionnaire’s validity. We asked six experts in CME (NM), medical education (TC and AH), psychology (ZD and IM) and e-learning (NZ) to review the questionnaire. Then, we distributed the questionnaire to four physicians in order to conduct cognitive testing. We asked them to read the questions and to comment if any of the questions were difficult to understand or did not make sense. We revised some items and scales to improve internal and face validity.

The final questionnaire had 25 items in four TPB dimensions. The translated version of the questionnaire is seen in Additional file 1. We measured participants’ beliefs with a 7-point bipolar scale ranging from completely disagree (−3) to completely agree (+3). Higher scores showed more positive opinions about e-Learning. Based on the TPB, our final questionnaire about using e-Learning in CME consists of five parts: intention, attitudes, subjective norms, perceived behavioral control and background factors (demographic items, and access to a computer and Internet).

### Distribution of the questionnaire

The data collection tool was a paper-based questionnaire. GPs who attended IUMS CME on site seminars in autumn 2014 were invited to participate in the study and, when informed consent was obtained, were asked to fill in a paper-based questionnaire and return it to the CME office. Verbally and also in the questionnaire’s cover letter, we informed them that the information would only be used for the purpose of the study and that filling out the questionnaire was voluntary. In order to increase the response rate, we provided a financial bonus deposited in their CME account (to be used to attend more CME courses) once they’ve returned a completed questionnaire.

The inclusion criteria were: show interest in participating in the study, return a complete questionnaire, be a GP and have eCME experience. The research ethics committee of IUMS approved this study protocol (293158).

### Statistical analysis

Most of our interval data and Likert items were quite normally distributed and had moderately acceptable kurtosis and skewness (below two and seven respectively) [21]. When needed, items were reverse-scored for better interpretation. We conducted an exploratory factor analysis using the principal component analysis method to evaluate the loading of variables. We used SPSS V.22 and Amos V.22 (IBM, Chicago, USA). With the exploratory factor analysis results, we executed confirmatory factor analysis and structural equation modeling by Amos in order to create measurement and structural models. Some of the Likert items had missing values (mostly for subjective norm items), and we imputed them via a regression model in Amos.

## Results

**Fig. 2:**
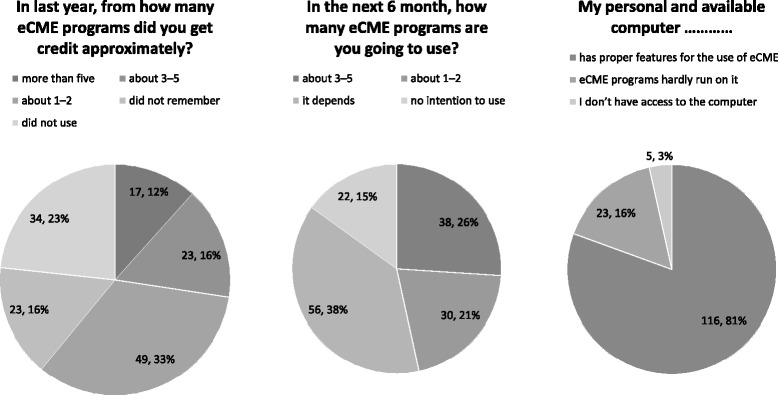
Demographic and background data of the participants

The questionnaire was distributed in seven CME seminars (with an average of 100 participants). One hundred forty eight GPs agreed to participate in the study and filled out the questionnaire. We excluded two incomplete questionnaires. Forty percent (*n* = 58) of the participants were female and the average age of the participants was 43 (from 28 to 72). Other demographic and background date were presented in Fig. 2.

We had three research questions (and tried to answer them in three parts of the results section): how do factors extracted by exploratory factor analysis match the theoretical constructs of TPB? (Dimensionality of the developed model), how does the statistical model of GPs’ intentions fit the data collected from an eCME setting? (Fitness of the model) and how does the model map GPs’ intention to use eCME? (predictability of the developed model).

### Dimensionality of the developed model

In this phase, by doing an exploratory factor analysis (EFA), we have created a model and evaluated dimensionality of this model with TPB’s theoretical components (first research question). For the EFA, we used the Principal Component Analysis method for extraction, the oblique method for rotation (Direct Oblimin, because most of the factors had a correlation above 0.3 [22] and the pattern matrix for the factor loading [23].

We excluded three items that had high skewness (>2) and kurtosis (>7) before the EFA [21]. Items were Q14 (working with computer/Internet, 2.40, 1.19, −2.76, 8.67), Q16 (personal computer features, 2.7, 0.49, −2.11, 3.72) and Q17 (computer skills, 2.29, 1.23, −2.20, 5.22) as mean, standard deviation, skewness and kurtosis.

In the primary EFA, we found five factors with an Eigen value higher than 1 and explained 58 % of the variance. Then, we limited the factor numbers to four (as TPB constructs and explained 54.1 % of the variance). The communalities for each item were sufficiently high (all above 0.3), indicating that the items were adequately correlated for a factor analysis (Table 1). Internal consistency for each factor was moderate to high, with the lowest Cronbach’s alpha at 0.56. For naming the factors, we considered the TBP constructs and also items with higher loadings in each factor [22].

**Table 1:** Pattern matrix for exploratory factor analysis of the questionnaire

Item	Factor 1 (PBC)	Factor 2 (SN)	Factor (Intention)	Factor 3 (Attitude)	Extraction communality
Q09f: eCME final exam	**.792**	-.036	-.209	.170	.652
Q18: eCME audiovisual	**.700**	-.006	.018	-.009	.498
Q09c: eCME scientific quality	**.677**	.013	.041	.125	.535
Q09b: eCME cost	**.630**	-.061	.180	-.172	.501
Q03: Improving practice	**.561**	.160	.306	.168	.560
Q09e: eCME Q&A	**.539**	-.203	-.058	-.144	.355
Q19: eCME & Internet speed	**.486**	-.100	-.019	.145	.330
Q08: Independent learning	**.410**	-.089	.283	.160	.462
Q10: Encouragement by boss	.069	**-.857**	-.067	.058	.757
Q11: Encouragement by CME office	-.082	**-.813**	.058	.031	.660
Q12: Encouragement by colleagues	.137	**-.768**	.080	-.059	.717
Q15: Concentrate with distractors	-.103	-.116	**.774**	.098	.661
Q06: eCME credit possibility	-.099	-.103	**.710**	.137	.578
Q20: CME preference	.250	-.011	**.708**	-.130	.650
Q02: Intention (next 6 month)	.366	.029	**.404**	-.034	.376
Q04: Traffic time	.066	.125	-.160	**.742**	.543
Q05: Job leave	-.056	-.107	.133	**.620**	.454
Q09a: eCME time saving	.227	-.043	.005	**.585**	.478
Q09d: More eCME credits	-.054	-.057	.108	**.529**	.315
Q07: Recommending	.388	-.101	.339	**.409**	.740
Cronbach’s alpha	.81	.8	.56	.78	
Eigen value	6.4	1.7	1.5	1.2	

Extraction Method: principal component analysis. Rotation method: Oblimin with Kaiser Normalization. Rotation converged in 12 iterations

Abbreviations: *PBC* perceived behavioral control, SN subjective norms

Bold numbers emphasize highest loadings in a column

The KMO measure of sampling adequacy was 0.837, and Bartlett’s test of sphericity was statistically significant (*p* < .01). The factors demonstrated sufficient discriminant validity. Factor correlation data for factors were: factor 1 (2, −0.32; 3, 0.35 and 4, 0.26), factor 2 (3, −0.32; 4, 0.11) and factor 3 (4, −0.24). We did not have any component correlation above 0.70, but they are still correlated, and we cannot assume them to be orthogonal. We removed Q13, because it had cross loadings.

### Fitness of the model

In order to arrive at the measurement model (second research question), we did a confirmatory factor analysis (CFA) using AMOS 22. Observed variables for each latent variable were acquired from the pattern matrix.

The factor loadings of the latent to observed variables was recommended to be higher than 0.3 [22]. The items’ standardized regression weights were greater than 0.3, and all of them had significant regressions with their related latent variables. This represents the amount of change in the latent variables that is attributable to a single standard deviation unit’s change in the item. We added covariance between two errors in the attitude’s observed variables. All of the modification indices were lower than ten. In order to check the model fit, we measured the indices presented in Table 2. We measured the CMIN (minimum discrepancy), which is similar to Chi-square. For an estimation of the average size of the residuals between actual covariance and the proposed model covariance, we used RMSEA. The recommended value also mentioned in last row [22]. Based on the result of CFA, we assigned Q07 to the intention factor.

**Table 2:** Goodness-of-fit indicators for model

Indices	Absolute fit indices			Incremental fit indices	Parsimony fit indices
	CMIN/DF	CMIN *p* value	RMSEA	CFI	PNFI
Current model	1.48	*p* < .01	0.06	*0.92*	0.68
Recommended value	1-2	*P* > .01	<0.10	*>0.95*	>0.50

Abbreviations: *CMIN* minimum discrepancy, *RMSEA* root mean square error of approximation, *CFI* comparative fit index, *PNFI* parsimony-adjusted normative fit index

### Predictability of the developed model

**Fig. 3:**
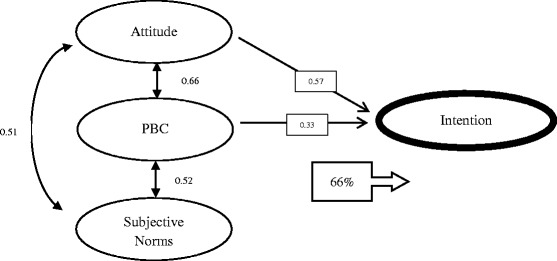
Predictability of main constructs of TPB to intention in our model. Numbers in the box are standardized regression weights and the Covariance among latent variables was presented near bidirectional arrows. All arrows had *p* < 0.01. The number in the arrowed box represents the intention’s variance which explains by 3 other latent variables. PBC indicates perceived behavioral control

In order to find the predictive power (third research question), we followed the structural equation modeling in our model using Amos. The Attitude and PBC to intention regression weights were statistically significant, but subjective norm was not, so we removed its regression arrow to intention. The explained variance of intention was 66 % (Fig. 3). The covariance among variables was presented. Regarding intention to use eCME in our participants, we did subgroup analysis for access to computer and internet and other demographic factors, but couldn’t find any statistically significant results.

## Discussion

Using the theory of planned behavior as a framework we developed an instrument to model GPs’ intention to use e-Learning in the context of continuing medical education. A number of studies about physicians’ attitude towards eCME exist and they focused on different areas like acceptance of eLearning in CME [8], comparison of CME and eCME [7], preferences and use of educational media [9] and perceived barriers (such as physicians ‘perceptions of time constraints, and unfamiliarity with computers) to completing eCME [10], but in order to explore the main factors, we need a theory-driven framework. As far as we could find in the literature, there is one study in eCME field that used theoretical concepts of TPB to make an evaluation instrument and not for modeling [24]. We followed standard steps in developing a TPB questionnaire and performed statistical analysis to check the questionnaire’s dimensionality, validity and predictability.

### Psychometric characteristics of the questionnaire

In the exploratory factor analysis, some of our designed attitudinal items cumulated in the PBC factor. The reason could be our definition of attitude and also the perception of the participants from the items as enablers or barriers. We had good and significant standardized regression weights for items with their related latent variables and also quite good explained variance with these four latent variables (higher than 50 % which is recommended) [22]. The subjective norm questions had the most missing values, which may indicate that our participants interpreted these items not as they were meant to, which was also the case in another study [25]. Also, we had the lowest covariance between this factor and other factors. Our eCME system (CD ROMS with online final exam) did not have a discussion forum, and GPs mostly use eCME without interaction with other users. As stated in another study [10], those may be the reasons that subjective norm could not explain the variance in eCME intention.

The recommended format of the TPB belief components consists of beliefs about the consequences of performing the behavior multiplied by the user’s evaluation of these consequences. But Ajzen questioned the Expectancy Value Theory of Fishebin [11],which reduces the psychological construct to a mathematical formula (attitude*weight). He mentioned that in some cases, removing the weighting part may increase the predictability power of the behavior. Technically, we had about 25 main questions; if we had wanted to measure the users’ evaluation part, then the questionnaire would have been too long for busy physicians.

### Modeling the eCME intention using TPB

Overall, this model demonstrated quite good fitness. Regarding the goodness-of-fit indicators, the RMSEA was good as an absolute fit index, although the Chi-square was not statistically significant. Low sample size affects this index [22]. The incremental fit index (CFI) was near the recommended value, indicating that a good correlation exists among variables in the study. Also, our model’s parsimony fit index was good enough.

The results indicated that he questionnaire could explain 66 % of the GPs’ intention to use eCME. Although the explained variance is context-specific [22], in a TPB study among GPs, researchers could explain 48 % of variation in reported intentions for following a specific guideline [26]. In most of the previous studies about technology acceptance among physicians, R-square was about 0.4, demonstrating that although a significant amount of physician intention is explained, some predictors of this intention remain unidentified [27].

We found that attitude and perceived behavioral control play a significant role in this model. Contrary to another study [28], access to computer and Internet had not a statistically significant role for predicting intention to use eCME in our participants.

### Limitations of the study

Our sample size was lower than optimum for factor analysis. On the other hand, about 20–25 % of all physicians in Isfahan use eCME (personal communication: Nahidossadat Mirshahzadeh, Director of CME office, IUMS). Then our response rate of 148, based on the CME population available in the context of study (175 out of 700 have eCME experience) is 83 % and it is satisfactory. Aiming at larger sample sizes would provide the possibility to perform more precise studies and also investigate subgroup analysis of physicians to find different predictors of intention. Such study could be conducted by using the developed questionnaire in this study and by following the TPB methodology.

## Conclusions

The findings demonstrate that the theory of planned behavior can help us understand the GPs’ intention to use eCME. The factors’ dimensionality was according to the TPB, and most of the items cumulated in theoretical factors. The model explaining the factors that lead to positive intention to use eCME also has quite good fit indices. The perceived behavioral control and attitudinal constructs were included in the model, but the subjective norms construct was not. Finally, we can explain 66 % of the intention’s variance using this model. The use of a TPB-based survey will increase the rigor of the research/evaluation and could support CME directors in assessing and improving GP’s intention to use eCME by adapting relevant variables based on this evidence.

## Additional file

Additional file 1: Translated questionnaire (eCME usage in general practitioners questionnaire). (PDF 430 kb)

## Abbreviations

CFA: Confirmatory factor analysis
CME: Continuing medical education
eCME: Electronic continuing medical education
EFA: Exploratory factor analysis
GP: General practitioners
IUMS: Isfahan University of Medical Sciences
PBC: Perceived behavioral control
SN: Subjective norms
TPB: Theory of planned behavior
