# Supplementary material for: Applicability of the theory of planned behavior in explaining the general practitioners eLearning use in continuing medical education
Source: BMC Med Educ. 2016 Aug 22;16(1):215. doi: 10.1186/s12909-016-0738-6 (PMC4994161; doi:10.1186/s12909-016-0738-6)
Supplement: Additional file 1: — Translated questionnaire (eCME usage in general practitioners questionnaire). (PDF 430 kb) [file 12909_2016_738_MOESM1_ESM.pdf]

In the name of God

Cover letter

Dear colleague,

If you are a GP and you used some e-Learning methods in CME (CD or online version), it is our pleasure to ask you spend some time and fill attached questionnaire.

Application of this research

In this study, we want to examine aspects of e-learning usage in CME. This study was supported by the Medical Education Research Centre in IUMS and Unit of Medical Education in Karolinska Institute. We hope with your help, we could use the results of the project for planning and implementation of online CME programs.

More information

Questionnaire data will be used only for this study. We need Your personal number to access your basic information and training scores in CME Office database to save your time (do not need to complete those items). If you require further information about this research, please email us to: arash.hadadgar@ki.se or z.dehghani88@gmail.com

Please don't spend more than 15 minutes of your time to answer the questions.

Please return the questionnaire up to the end of the June 2014 to the CME Office, and ask them to sign this page. To appreciate the thorough completion of the questionnaire, the cost of an online CME (3 dollars) will be charged to your CME account (according to your personal number in the questionnaire). Thanks for your time,

Please detach this sheet from the questionnaire and keep it.

CME office signature

It is a translated version of the questionnaire and may contain linguistic errors.

eCME usage in general practitioners questionnaire

Personal code	Name	program	Questionnaire code

What is this research about?	Dear colleague, as you know, by using information and communication technologies, we could improve the education. Because the production of the eCME programs is expensive, it is necessary to understand the skills and preferences of the physicians and also the barriers in this field. This study aims to do so.
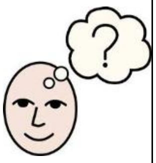 How to answer?	The questions are about your experience in using elearning in CME (CD and online version). If you have such an experience and you are a GP, then please fill the questionnaire. Please think more about your eCME experience, and then start to answer the questions. Filling this questionnaire is completely voluntary and your personal information will be protected in this study. Your honest answers will help to tailor future eCME programs to the users' interest and skills. For the attitudinal questions, please specify the place which is closer to your opinion, for example: Completely disagree ---(-3)---(-2)---(-1)---(0)---(+1)---(+2)---(+3)--- completely agree

1- In <u>last year</u> (2013), from how many eCME programs did you get credit approximately:

(--) more than 5 programs (--) 3-5 programs (--) 1-2 programs (--) didn't use (--) don't remember

2- In the <u>next 6 month</u>, how many eCME programs are you going to use:

(--) 3-5 programs (--) 1-2 programs (--) not decided yet or it depends (--) will not use

3- Using eCME programs help me to improve my practice:

Completely disagree ---(-3)---(-2)---(-1)---(0)---(+1)---(+2)---(+3)--- completely agree

4- Elimination of travel time and traffic is a major factor in my use of eCME:

Completely disagree ---(-3)---(-2)---(-1)---(0)---(+1)---(+2)---(+3)--- completely agree

5- No need to leave the job, is one of my reasons for using eCME:

Completely disagree ---(-3)---(-2)---(-1)---(0)---(+1)---(+2)---(+3)--- completely agree

6- It is better to provide the possibility to earn more credits through eCME:

Completely disagree ---(-3)---(-2)---(-1)---(0)---(+1)---(+2)---(+3)--- completely agree

7- What is your recommendation to your colleagues about eCME:

I do not recommend to use eCME	---(-3)---(-2)---(-1)---(0)---(+1)---(+2)---(+3)---	As far as possible make use of eCME

8- The experience of independent learning in eCME method, for me.................

(--) has been a pleasant (--) has failed (--) has been unpleasant (--) I think it is too early for the medical community

9- Please help us to know more about your experience from eCME:

Have you spent on eCME program, what you think of the following?

a	In terms of time saving	Valuable ---(-3)---(-2)---(-1)---(0)---(+1)---(+2)---(+3)--- Time consuming
b	In terms of costs	Convenient ---(-3)---(-2)---(-1)---(0)---(+1)---(+2)---(+3)--- Too much
c	In terms of scientific quality	Good ---(-3)---(-2)---(-1)---(0)---(+1)---(+2)---(+3)--- Bad
d	The opportunity to earn CME credits	Easy ---(-3)---(-2)---(-1)---(0)---(+1)---(+2)---(+3)--- Hard
e	In terms of possible Q&A with teachers	Possible ---(-3)---(-2)---(-1)---(0)---(+1)---(+2)---(+3)--- Impossible
f	In terms of final exam questions:	Applied ---(-3)---(-2)---(-1)---(0)---(+1)---(+2)---(+3)--- not applicable

10-My bosses are encouraging me to use the eCME (if you work in a hospital or medical clinic, please answer this question)

Completely disagree ---(-3)---(-2)---(-1)---(0)---(+1)---(+2)---(+3)--- completely agree

11-Manager and staff of the university CME office, are encouraging me to use the eCME:

Completely disagree ---(-3)---(-2)---(-1)---(0)---(+1)---(+2)---(+3)--- completely agree

12-My GP friends are encouraging me to use the eCME:

Completely disagree ---(-3)---(-2)---(-1)---(0)---(+1)---(+2)---(+3)--- completely agree

13-CME rules and regulations are encouraging me to use the eCME:

Completely disagree ---(-3)---(-2)---(-1)---(0)---(+1)---(+2)---(+3)--- completely agree

14-Today, working with computer and the Internet is inevitable for physicians:

Completely disagree ---(-3)---(-2)---(-1)---(0)---(+1)---(+2)---(+3)--- completely agree

15-Distraction agents in the online environment (email, and social networks), prevent me to focus on eCME:

Completely disagree ---(-3)---(-2)---(-1)---(0)---(+1)---(+2)---(+3)--- completely agree

16-My personal and available computer …………

(--) has proper features for the use of eCME (--) eCME programs hardly run on it (--) I don't have access to the computer

17-What is your situation in using computer for eCME:

I am not ready to work with computers for eCME	---(-3)---(-2)---(-1)---(0)---(+1)---(+2)---(+3)---	I do all computer works of eCME by myself

18-According to audiovisual principles, most of the educational materials and multimedia in eCME are …………….

monotonous and exhausting	---(-3)---(-2)---(-1)---(0)---(+1)---(+2)---(+3)---	engaging and encourage learning

19- According to the usual Internet speed, most of the eCME programs are run and downloaded well:

Completely disagree ---(-3)---(-2)---(-1)---(0)---(+1)---(+2)---(+3)--- completely agree

20-Between two supposed <u>online</u> and <u>seminar</u> CME programs, with equal quality and same subject, which one do you prefer:

Online --◄( )——( )——( )——( )——( )——( )——( )►-- Seminar

In order to understand which kind of GPs could take more advantages of the eCME, we need some more information about your practice and interaction with eCME. Any information that you think is too personal, Please do not complete answer it!

21-Please specify your clinical workplace (if it is necessary, select more than one option):

(--) Private office (--) Clinic/hospital University (--) Working at university administration (--) Now, I do not practice

22-In the home and work, how much do you use computer?

A) **Home**: (--) Daily (--) Weekly (--) Monthly (--) I have access to computer but I don't use it (--) I don't have access to computer

B) **Work**: (--) Daily (--) Weekly (--) Monthly (--) I have access to computer but I don't use it (--) I don't have access to computer

23- In the home and work, what is your dominant method of internet connection?

A) **Home**: (--) ADSL (--) WiMAX (--) Dial up (--) I don't know (--) I don't have access to internet (--) I don't have access to computer

A) **Work**: (--) ADSL (--) WiMAX (--) Dial up (--) I don't know (--) I don't have access to internet (--) I don't have access to computer

24-How frequently do you use your email?

(--) Daily (--) Weekly (--) Monthly (--) I forgot my email password (--) I don't have email address (--) I don't have access to computer and internet

If you would like to be informed of the results of the project, please mention your email here:

. . . .

In the meantime, thanks for your attention; we will try use the results of this project in design and implementation of the future eCME. If you have any tips or advice in the field of eCME please write them here:

. . . .

. . . .

. . . .

. . . .
